# Longitudinal microarray analysis of cell surface antigens on peripheral blood mononuclear cells from HIV+ individuals on highly active antiretroviral therapy

**DOI:** 10.1186/1742-4690-5-24

**Published:** 2008-03-04

**Authors:** Jing Qin Wu, Wayne B Dyer, Jeremy Chrisp, Larissa Belov, Bin Wang, Nitin K Saksena

**Affiliations:** 1 Retroviral Genetics Division, Center for Virus Research, Westmead Millennium Institute, University of Sydney, Darcy Road, Westmead, NSW 2145, Sydney, Australia; 2 Immunovirology Laboratory, Australian Red Cross Blood Service, Clarence Street, Sydney, NSW 2000, Australia; 3 Medsaic Pty Ltd, Suite 145, National Innovation Centre, Australian Technology Park, Garden Street, Eveleigh, NSW 1430, Sydney, Australia

## Abstract

**Background:**

The efficacy of highly active antiretroviral therapy (HAART) determined by simultaneous monitoring over 100 cell-surface antigens overtime has not been attempted. We used an antibody microarray to analyze changes in the expression of 135 different cell-surface antigens overtime on PBMC from HIV+ patients on HAART. Two groups were chosen, one (n = 6) achieved sustainable response by maintaining below detectable plasma viremia and the other (n = 6) responded intermittently. Blood samples were collected over an average of 3 years and 5–8 time points were selected for microarray assay and statistical analysis.

**Results:**

Significant trends over time were observed for the expression of 7 cell surface antigens (CD2, CD3epsilon, CD5, CD95, CD36, CD27 and CD28) for combined patient groups. Between groups, expression levels of 10 cell surface antigens (CD11a, CD29, CD38, CD45RO, CD52, CD56, CD57, CD62E, CD64 and CD33) were found to be differential. Expression levels of CD9, CD11a, CD27, CD28 and CD52, CD44, CD49d, CD49e, CD11c strongly correlated with CD4+ and CD8+ T cell counts, respectively.

**Conclusion:**

Our findings not only detected markers that may have potential prognostic/diagnostic values in evaluating HAART efficacy, but also showed how density of cell surface antigens could be efficiently exploited in an array-like manner in relation to HAART and HIV-infection. The antigens identified in this study should be further investigated by other methods such as flow cytometry for confirmation as biological analysis of these antigens may help further clarify their role during HAART and HIV infection.

## Background

In our recent study, we have used the DotScan antibody microarray technology to identify differential cell surface markers expressed on CD4+ and CD8+ T cells between 3 HIV disease groups and uninfected controls [1]. Along with confirming the cell surface markers previously described in the context of HIV disease, we identified 5 novel markers that could segregate HIV disease stages. This study together with the study by Woolfson et al., who used a similar antibody microarray to show the conservation of unique cell surface antigen mosaics in cryopreserved PBMCs from HIV+ individuals [2], demonstrated the power of this technology as an adjunct to flow cytometry in HIV research. Even though T cell subsets could provide more specific information, as evident from our previous study [1], PBMCs have already been shown to be acceptable as starting material for antibody microarray analysis of HIV disease status as well as for classifying leukemia types [2,3].

During the natural course of HIV infection, the major determinant of the depletion of CD4+ T cells is immune activation [4]. Several previously described surface markers are up-regulated on T cells during the activation process, and are known to have a profound effect on the course of HIV disease [4]. Importantly, progression of HIV infection correlates with increases in circulating markers of immune activation such as soluble interleukin-2 receptors (sIL-2R) [5], soluble tumor activation markers such as necrosis factor receptor type II (sTNF-RII) [6] and monocyte activation markers such as neopterin [7]. Recently, a few new cell surface markers involved in HIV pathogenesis and disease progression have been identified. These include CD137L (4-1BBL), which was shown to be a critical component in the rescue of functionally impaired HIV-specific CD8+ T cells [8]; CTLA-4, the inhibitory immunoregulatory receptor, whose expression correlated positively with disease progression and negatively with the capacity of interleukin 2 production by CD4+ T cells in response to viral antigen [9]; and PD-1 on HIV-specific T cells, the inhibitory receptor programmed death 1, whose expression was associated with T-cell exhaustion and disease progression [10].

The advent of HAART has led to a dramatic decline in AIDS-related morbidity and mortality by decreasing plasma viremia and increasing CD4+ T cell counts [11,12], normalizing the progenitor cell function [13] and restoring CD4+T-cell functions [14,15]. In treatment-naive individuals who initiate HAART and can attain complete viral suppression, T cell activation declines as plasma viremia decreases [16]. Treatment failure appears to be associated with increases in T cell activation and rapid decline in CD4+ T cell numbers. In contrast, T cell activation appears to decrease in patients attaining good control of viral replication while on HAART, and is maintained at low levels during the prolonged periods of complete viral suppression [17]. In some patients achieving suppression of viremia, T cell activation may still be evident. This may be attributable to residual viral replication, and this may affect the extent of CD4+ T cell recovery during HAART. Although HAART's ability to reduce viral load to below the detection levels has been well documented, the mechanisms involved in the immune reconstitution resulting from this treatment are still not fully understood. A thorough characterization of changes induced by HAART on the broad immunenophenotype of the immune cells over time may facilitate the clarification of these mechanisms.

Although a considerable amount of work has already been done to elucidate surface marker modulation during HIV disease and therapy by flow cytometry, this study is the first to use a cell-based antibody microarray (135 antigens) to retrospectively and longitudinally monitor the effect of antiretroviral therapy on cell surface antigen expression using frozen PBMC over time. Two HIV+ groups were studied: sustained responders (SR) who achieved sustainable response by maintaining below detectable plasma viremia on HAART and transient responders (TR) who responded intermittently to HAART. Our hypothesis is that modulation of cell surface markers occurs during the course of HIV disease and following the initiation of HAART and these cell surface markers may indicate the outcome of antiretroviral therapy. Along with confirming the cell surface markers previously described, we aimed at identifying novel potential cell surface markers associated with HIV disease progression and HAART efficacy.

## Methods

### Patient profiles

**Figure 1:**
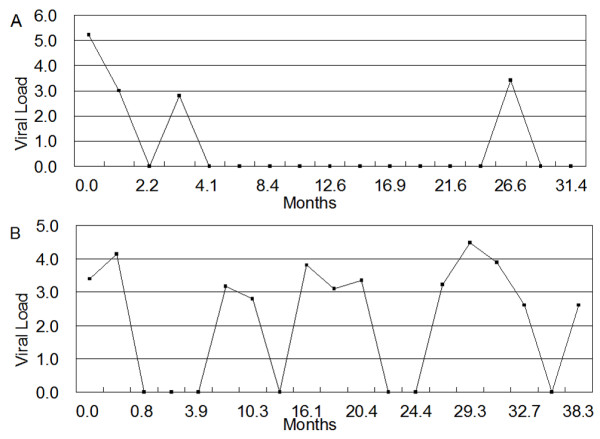
Representative viral load plots for (A) sustained responder and (B) transient responder. Log_10 _of HIV RNA copies/ml in plasma, detected by quantitative reverse transcription-PCR, was plotted against time from the date of initiation of therapy. Values of HIV RNA copies/ml below the detection level are shown as zero.

This study was approved by the Sydney West Area Health Services Research Ethics Committee and all blood samples were obtained upon written informed consent from each patient. Twelve HIV+ patients were enrolled from Sydney, Australia and blood samples were collected over an average of 3 years with 33 time points on average for each patient. Five to eight time points were chosen according to the duration of the therapy usage for microarray assay and correlation analysis. The 4 time points that had similar duration of therapy for each patient were further selected for studying time related changes: (1) the initiation date of the therapy; (2) during the first year of therapy; (3) between 1 year and 1.5 years after therapy; (4) ≥ 2 years after therapy. At each time point, the CD4+ and CD8+ T cell counts as well as the plasma viral loads were measured (Table 1). Based on the virological response to HAART, the HIV+ patients were stratified into two groups: sustained responders (SR; n = 6) and transient responders (TR; n = 6). Within the sustained responder group, the time points with detectable viral load for each patient were 0–6% of the total points collected. One patient had no detectable viral load throughout the therapy, 4 patients achieved successful suppression of plasma viral load from the baseline to below detection levels and maintained at all time points except one time point with viral load < 1000 copies/ml, and one patient had 2 time points with low detectable viral load and this patient's viral load kinetics is shown in Figure 1A as an example. In the case of transient responders, plasma viral load was controlled to below detectable levels only intermittently, each patient had 30–70% time points showing variable plasma viral loads. For illustration, one patient's viral load kinetics is shown as a representative (Figure 1B). Patients received combination antiretroviral therapy, which included: zidovudine, didanosine, stavudine, lamivudine, nevirapine, indinavir, ritonavir, nelfinavir and/or saquinavir. All the patients received at least two reverse transcriptase inhibitors in association with one protease inhibitor except two patients who received combined therapy of non-nucleoside reverse transcriptase inhibitors and nucleoside analogs without protease inhibitor. For comparison, control samples from 23 HIV-negative individuals were also analyzed.

**Table 1:** Patient characteristics

Parameter	Group	Baseline	Time point 2	Time point 3	Time point 4
CD4 counts^c^	SR^a^	820 (800–1050)	880 (800–960)	1050 (978–1228)	1000 (825–1145)
	TR^b^	560 (480–720)	705 (588–887)	673 (557–835)	782 (627–903)
CD8 counts^c^	SR^a^	950 (780–1140)	1029 (828–1175)	824 (705–960)	890 (675–992)
	TR^b^	1200 (1040–1300)	915 (819–979)	969 (880–1062)	918 (862–967)
Viral load^d^	SR^a^	3 BDL (169841–750000)	4 BDL (624, 931)	5 BDL (810)	6 BDL
	TR^b^	3 BDL (2500–58292)	4 BDL (1340, 95280)	3 BDL (1300–8146)	1 BDL (390–180991)

a SR: sustained responder group.

b TR: transient responder group.

c CD4 and CD8 counts were median (first quartile-third quartile), expressed as cell numbers per μl blood.

d Viral load shows the number of patients with below detectable level (BDL) of virus; also shown, in brackets, are the HIV-1 copy numbers/ml for the viremic patients, with a range of the HIV-1 copy numbers shown for groups with more than 2 viremic patients.

### Antibody microarray construction

Medsaic Pty. Ltd. (Eveleigh, NSW, Australia) provided the DotScan™ microarrays, prepared as previously described [3]. Monoclonal antibodies were purchased from the following companies: Coulter and Immunotech from Beckman Coulter (Gladesville, NSW, Australia), Pharmingen (BD Biosciences, North Ryde, NSW, Australia), Biosource International (Applied Medical, Stafford City, QLD, Australia), Serotec (Australian Laboratory Services, Sydney, NSW, Australia), Sigma-Aldrich (Castle Hill, NSW, Australia), Biotrend, Biodesign and MBL (Jomar Diagnostics, Stepney, SA, Australia), Chemicon Australia (Boronia, VIC, Australia), Leinco Technologies (St. Louis, MO, USA) and Calbiochem (Merck, Kilsyth, VIC, Australia). Antibody solutions were reconstituted as recommended, and stored in aliquots with 0.1% (w/v) BSA at -80°C; Pharmingen antibodies were generally stored at 4°C. Antibodies were used for making microarrays at concentrations ranging from 50–1000 μg protein/ml.

### Immunophenotyping of PBMC

**Figure 2:**
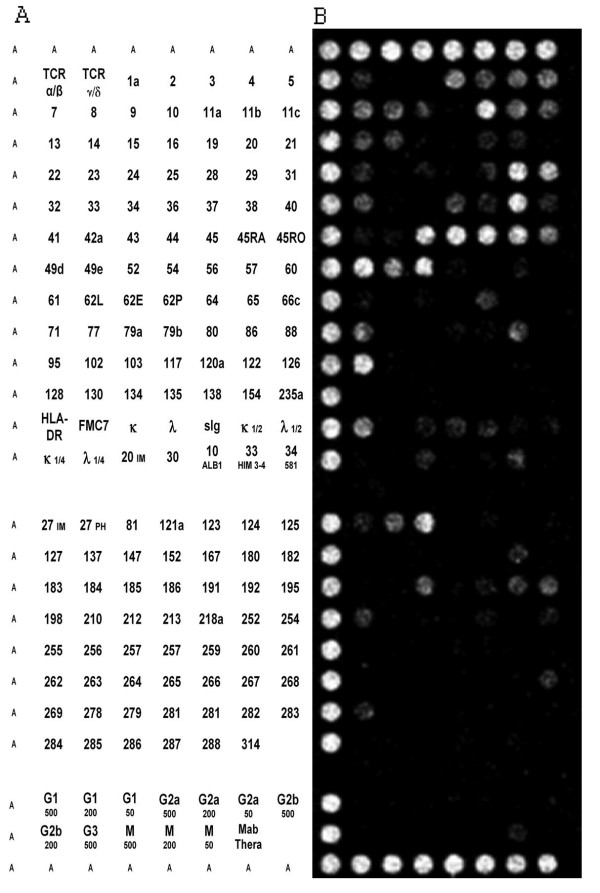
Composite dot scan patterns of antibody binding for PBMC cells. Half of the duplicate array was shown with the alignment dots "A" at left, top and bottom. Alignment dots are a mixture of CD44 and CD29 antibodies. (A) The key for CD antigens on the DotScan array and (B) Representative patient PBMC binding pattern.

Mononuclear cells were purified by Ficoll density gradient centrifugation and cryopreserved in fetal calf serum (FCS) with 10% dimethylsulfoxide (Sigma, Poole, United Kingdom). The cryopreserved cells were rapidly thawed and washed in PBS and the viability was examined using trypan blue dye exclusion method. Cell populations were then tested on antibody microarrays using DotScan technology as previously described [18]. Briefly, 4 × 10^6 ^cells were suspended in 300 μl PBS with added heat-inactivated human AB serum and the cell suspension was incubated for 40 minutes on the microarray chip, after which unbound cells were removed by gentle immersion in PBS. Captured cells were fixed in 3.7% (w/v) formaldehyde and imaged using a Medsaic DotReader™. Dot intensities were quantified for each antibody in duplicate using Dot Scan data analysis software on an 8-bit pixel grey scale from 0–255. The dot intensity reflects cell binding density, which depends on both the level of expression of a particular antigen and the proportion of cells expressing that antigen [18]. The dot pattern obtained is the immunophenotype of that population of leukocytes (Figure 2).

### Statistical derivations

Sustained and transient responder groups had been established a priori. Data were log transformed before analysis to stabilize variances and improve normality. Following transformation, the distributional properties for individual antibodies were examined using box plots and kernel density estimators.

Time related changes of antibody expression were analyzed using repeated measures mixed model analysis of variance, with subject as a random effect. Time, group and time by group interaction were treated as fixed effects.

The relationship between antibody expression and CD4+ or CD8+ T cell counts were evaluated using repeated measures mixed model analysis of covariance. Subject was regarded as a random factor. Group, CD4+ or CD8+ T cell counts and group by CD4+ or CD8+ T cell counts interaction were regarded as fixed effects.

Parameter estimates were obtained using the REML algorithm [19]. Computations were performed using the techniques of Pinheiro and Bates [20]. Each antibody was analyzed separately, p values were adjusted using Holm's method [21], a conservative approach to maintain strong control of the family wise type I error rate.

## Results

### Antigens whose expression level showed a trend over time common to both HIV+ groups

**Figure 3:**
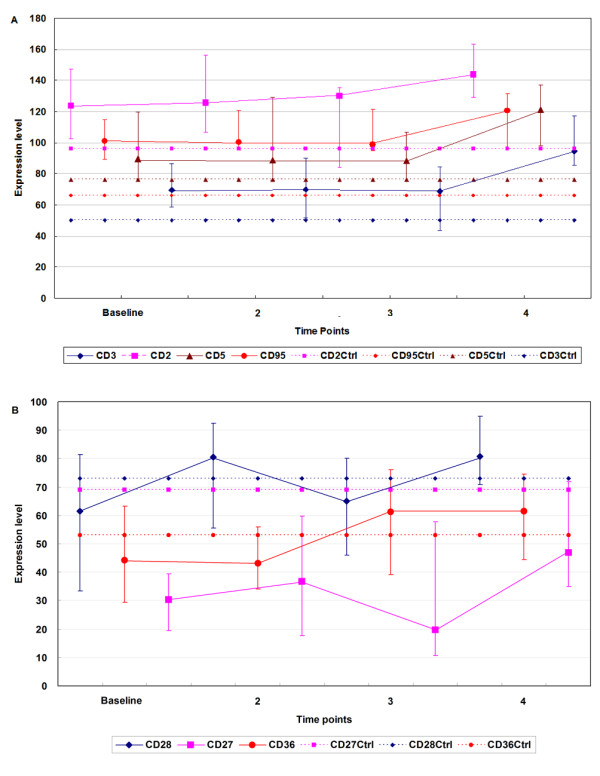
Time related changes in the PBMC cell surface antigens in HIV patients on HAART: (A) CD2, CD3epsilon, CD5 and CD95; (B) CD27, CD28 and CD36. Median cell binding values are linked by solid lines; bars indicate the 25^th ^and 75^th ^quartile values. The mean binding value of healthy controls at a single time point is represented by a dashed line. Time points were: (1) the initiation date of therapy; (2) within the first year of therapy; (3) at 1 to 1.5 years; and (4) at ≥ 2 years. To avoid the overlapping, the bars representing each antigen were staggered at each time point.

All 12 patients from both SR and TR groups were included to derive common trends in surface marker expression levels over time using repeated measures mixed model analysis of variance. The trends from baseline (time point 1) to time point 4 were significant for 7 cell surface antigens (Table 2). CD2 expression increased significantly from a baseline median of 124 to 144 at time point 4 (p = 0.047). Over the same time period, CD3epsilon (component of T cell receptor) expression increased from a median of 70 to 94 (p = 0.007), CD5 expression increased from a median of 90 to 121 (p = 0.04), and CD95 expression increased from a median of 101 to 121 (p = 0.032). A major change was noted in CD36 expression (p = 0.017) at time point 3 (1–1.5 years after therapy), whereas the expression of CD27 (p = 0.015) and CD28 (p = 0.007) fluctuated during the treatment period. Trends over time for the expression level of these antigens are shown in Figure 3. For reference, the average expression levels of the above antigens from 23 HIV negative controls at a single time point were also included in figure 3. The average values of dot intensity of CD2, CD3, CD5, CD95, CD27, CD28 and CD36 were 96, 50, 76, 66, 69, 73 and 53, respectively.

**Table 2:** Changes over time in the expression of cell surface antigens (p < 0.05) on PBMC from HIV+ individuals treated with highly active antiretroviral therapy

Antigen	Baseline	TimePoint2	TimePoint3	TimePoint4	P value^a^
CD2	124 (103–147)	126 (107–156)	130 (84–136)	144 (129–163)	0.047
CD3	70 (59–86)	70 (52–90)	69 (43–85)	94 (85–117)	0.007
CD5	90 (76–120)	89 (76–129)	88 (75–106)	121 (98–137)	0.040
CD36	44 (29–63)	43 (34–56)	61 (40–77)	62 (45–75)	0.017
CD95	101 (89–115)	100 (95–121)	99 (95–121)	121 (97–131)	0.032
CD27	30 (19–40)	37 (18–60)	20 (11–57)	47 (35–72)	0.015
CD28	61 (33–81)	81 (55–92)	65 (47–80)	81 (71–95)	0.007

Data are presented as median dot intensities (i.e., cell binding densities) quantified using DotScan data analysis software on an 8-bit pixel grey scale from 0–255. The 25^th^–75^th ^quartile values are shown in brackets. The cut off value of 10 was used for the detectable expression levels.

a Level of significance of the time parameter in a repeated measure mixed model analysis of variance was used to assess the changes in cell binding density values over time.

### Antigens discriminating between sustained and transient responders

**Figure 4:**
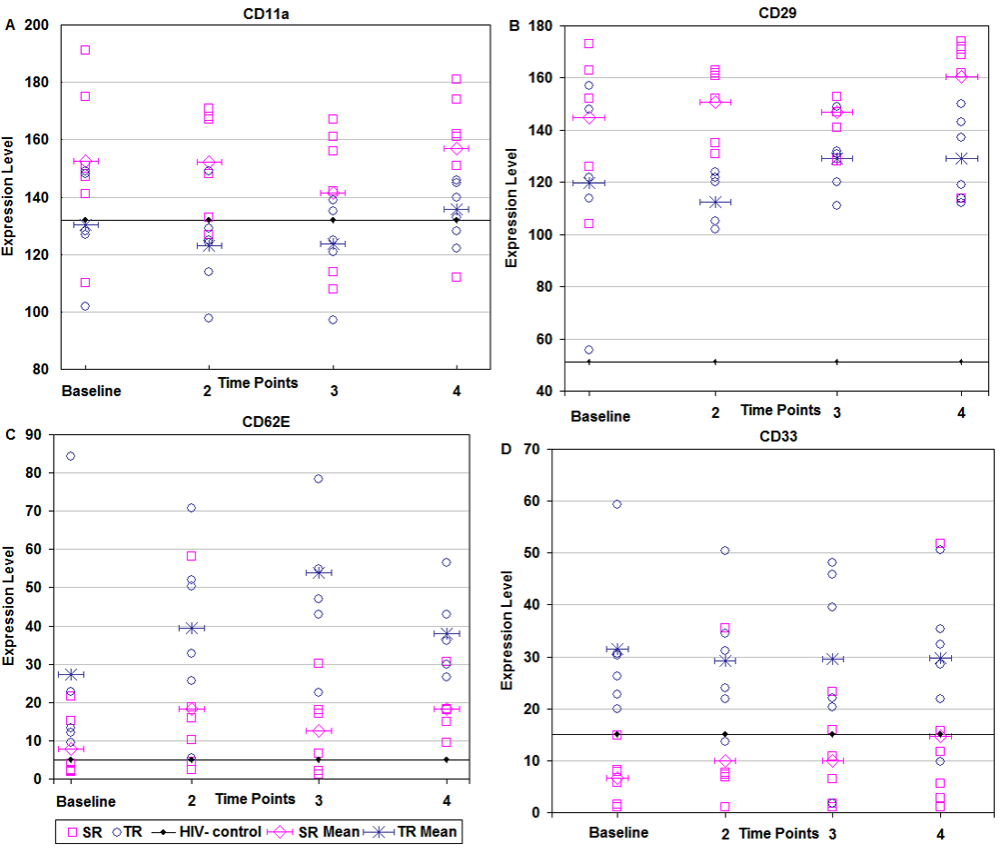
Comparison of (A) CD11a,(B) CD29, (C) CD62E and (D) CD33 expression in sustained responder (SR) and transient responder (TR) to HAART. (A) and (B) showed significantly higher levels in SR than in TR. (C) and (D) showed significantly lower levels in SR than in TR. Squares in pink and circles in blue represent cell binding density values for 6 SR and 6 TR patients, respectively, at time points 1 (baseline), 2, 3 and 4 (Time points as described in Figure 3). Bars with diamond and star symbols represent the mean values for the SR and TR groups, respectively, while the solid line with dots represents the mean value for the healthy controls at a single time point. The cut off value of 10 was used to identify detectable expression levels.

The repeated measures mixed model analysis of variance also identified antigens discriminating between sustained and transient groups. The expression of CD11a, CD29, CD38, CD45RO and CD52 was significantly higher at all time points in the sustained responder group as compared to the transient responder group, with p values ranging from 0.001 to 0.048 (Table 3); results for CD11a and CD29 are shown in Figure 4A and 4B, respectively. For reference, the average dot intensities of CD11a and CD29 (132 and 51, respectively) from negative controls were also included in the figure. In contrast, the expression of CD56, CD57, CD62E, CD64 and CD33 was significantly lower at all time points in the sustained responder group compared to the transient responder group, with p values ranging from < 0.001 to 0.047 (Table 3). Figure 4C and 4D show the difference between the SR and TR groups on the basis of CD62E and CD33 expression, respectively. For reference, the average dot intensities of CD62E and CD33 (5 and 15, respectively) from negative controls were also included in the figure.

**Table 3:** Antigens discriminating between sustained and transient responders

**Part A Antigens with significantly higher expression in SR compared to TR**					
Antigen	Baseline	TimePoint2	TimePoint3	TimePoint4	P value^a^

CD11aSR^b^	153 ± 28.1	152 ± 19.2	141 ± 25	157 ± 24.4	0.002
CD11aTR^c^	130 ± 17.2	123 ± 17	124 ± 14.7	136 ± 9.6	
CD29SR	145 ± 25.5	151 ± 14.3	147 ± 20.3	160 ± 23.1	0.001
CD29TR	120 ± 35.5	113 ± 10.5	129 ± 12.8	129 ± 16.2	
CD38SR	119 ± 32.4	123 ± 13.7	131 ± 21.3	138 ± 19	0.045
CD38TR	105 ± 19.3	106 ± 28.4	109 ± 16.4	116 ± 20.1	
CD45ROSR	107 ± 38.8	107 ± 17.9	105 ± 24.2	108 ± 19.2	0.003
CD45ROTR	72 ± 18.7	75 ± 17.5	64 ± 27.1	73 ± 30.5	
CD52SR	127 ± 40.5	136 ± 15.9	136 ± 22.5	139 ± 10.9	0.048
CD52TR	112 ± 25.1	111 ± 19.6	114 ± 21.3	118 ± 19.3	

**Part B Antigens with significantly lower expression in SR compared to TR**					

Antigen	Baseline	TimePoint2	TimePoint3	TimePoint4	P value^a^

CD56SR^b^	0	17	17	17	0.047
CD56TR^c^	17	33	33	83	
CD57SR	33	50	17	50	0.022
CD57TR	83	67	83	83	
CD62ESR	33	67	50	83	0.012
CD62ETR	83	83	100	100	
CD64SR	67	83	83	67	0.012
CD64TR	100	100	83	100	
CD33SR	17	17	50	50	< 0.001
CD33TR	100	100	83	83	

Part A: Data presented are dot intensities, shown by mean ± standard deviation.

Part B: Data are presented as the percentage of patient samples showing detectable expression level of the corresponding antigens in each patient group since some samples had below detection expression levels for some antigens. The cut off value of 10 was used to identify detectable expression levels.

a Level of significance of the group parameter in a repeated measure mixed model analysis of variance was used to assess the difference in values between groups.

b SR: sustained responder group.

c TR: transient responder group.

### Antigen expression correlated with CD4+ or CD8+ T cell counts

**Figure 5:**
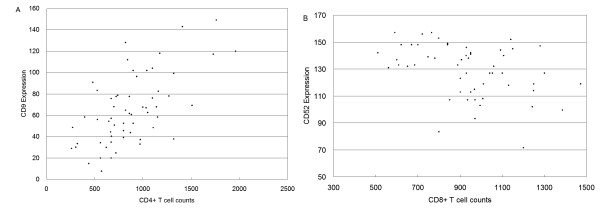
Correlation of (A) CD4+ T cell counts with CD9 expression and (B) CD8+ T cell counts with CD52 expression during HAART. Cell counts are expressed as cell numbers per μl blood, expression represents dot intensities (i.e., cell binding density) quantified using DotScan data analysis software on an 8-bit pixel grey scale from 0–255. Data was combined from all time points selected for microarray assay.

The correlation between CD4+ or CD8+ T cell counts and the density of PBMC binding on antibodies specific to 135 cell surface antigens was evaluated using a repeated measure mixed model analysis of covariance. CD9, CD11a, CD27 and CD28 showed a strong positive correlation with CD4+ T cell counts (p ≤ 0.001), while CD52, CD44, CD49d, CD49e and CD11c showed a strong negative correlation with CD8+ T cell counts (p = 0.003). Figure 5A shows CD9 expression against CD4+ T cell counts, while Figure 5B shows CD52 expression against CD8+ T cell counts.

## Discussion

Using DotScan technology, we have recently carried out a cross-sectional study to demonstrate that HIV disease stages can be segregated by cell surface antigens on CD4+ and CD8+ T cells [1]. The present study, to our knowledge, is the first retrospective longitudinal study using antibody microarray to monitor the effect of HAART based on CD marker expression using frozen PBMC. Simultaneous analysis of 135 cell surface antigens on PBMC from 12 HIV+ patients on HAART was performed over a two year period, and the patients were stratified into sustained responders and transient responders. Our study not only demonstrated potential associations between modulations of cell surface antigens and activation or restoration of the immune system, but also identified markers segregating sustained and transient responders to antiretroviral therapy, as well as markers significantly correlating with CD4+ or CD8+ T cell counts.

The majority of antigens which showed a trend over time for combined patient groups were associated with cell activation, implicating a general immune activation status of PBMC from patients on HAART. Notably, this activation was initially controlled during the first year, but was ultimately elevated after two years of HAART therapy. For instance, after 2 years of HAART, the CD3epsilon, and two co-stimulatory molecules CD27 and CD28 were upregulated relative to the baseline after an initial period of stability for the first 12–18 months. In HIV-infected individuals, the primary signal through TCR/CD3 is decreased, though response to costimulation through CD27 and CD28 is relatively preserved [22]. These stimulatory signal receptors were all increased during HAART, possibly as a consequence of the partial restoration of the impaired T cell responses during HAART.

CD95, CD2 and CD5 also showed a pattern similar to what was observed for CD3epsilon. The increase in CD95 expression over time was consistent with a previous study, which showed lack of control of T cell apoptosis under HAART [23]. CD2 mediates both cell-to-cell adhesion and T cell activation; also the CD2/LFA3 pathway may cooperate with signals from the TCR pathway to amplify HIV expression *in vivo *[24]. The biological relevance of increased CD5 expression is unclear though it has been suggested that up-regulation of CD5 on T cells can be a physiological event depending on protein kinase C activation [25]. Alternatively, the increase in CD5 binding may reflect the restoration of CD5+ T cell numbers in HAART treated individuals. Although HAART prolongs the period of controlled T cell activation, the observed elevation of activation markers over time indicates the eventual failure of HAART to control the chronic immune activation caused by HIV infection. It is thus plausible to hypothesize that during the initial stage of HAART therapy (up to 1.5 years in our study), substantial decreases in HIV antigen lead to the transient lowering of immune activation. However HAART eventually fails to control low level replication in HIV reservoirs, which is possibly responsible for continued cellular activation at the later stages of therapy, even when the viral load remains below detection.

The demonstrated increases in CD36 expression over time may be associated with the lipid metabolism derangements caused by HAART. In support of our findings, which showed increase in CD36 expression over time during HAART, Dressman et al., [26] showed that CD36 plays a crucial role in cellular uptake and accumulation of lipids, and protease inhibitors induce a specific increase in macrophage CD36 levels, which may promote accumulation of sterol in macrophages, foam cell formation and atherosclerosis [26]. Increased CD36 expression has also been found on circulating monocytes during HIV infection, which could represent a proatherogenic condition in HIV-infected patients [27]. Although the mechanisms regulating CD36 expression during HIV infection and HAART remains to be elucidated, it is imperative to carefully evaluate the role of CD36 expression especially during HAART as this treatment is known to be associated with increased cardiovascular risk, hyeprlipidemia and lipodystrophy in HIV patients. Surprisingly we didn't observe any statistically significant trend over time for CD4 and CD8 expression. But compared to the pre-therapy time points, the median of CD4+ T cell counts increased slightly while CD8+ T cell counts decreased slightly (Table 1). The lack of significant trend may be due to too many overlapping values in the cell counts between the time points and this trend may be detected by enlarging the sample size and increasing the time points.

Our study also found that five cell adhesion molecules (CD11a, CD11c, CD44, CD49d, CD49e) might serve as surrogate markers for disease progression, since the changes in expression levels of these molecules were highly correlated with the changes of either CD4+ or CD8+ T cell counts (p < 0.001). To our knowledge, this is the first report of a clear correlation between adhesion molecules and CD4+ and CD8+ T cell counts, though altered CD11a, CD44 and CD49e expression on cell subsets during HIV infection or disease progression has previously been reported [28,29]. Although the biological significance of the adhesion molecules remains largely unknown, it has been suggested that the plasma levels of several soluble adhesion molecules (CD11b and CD54) may have a potential application in assessing prognosis and efficacy of the HAART [30]. Therefore, the relationship between patient response to HAART, cell surface expression of adhesion molecules and levels of circulating adhesion molecules requires further investigation.

Three cell surface antigens were associated with cell activation (CD9, CD27 and CD28) positively correlated with the CD4+ T cell counts. Previous studies may provide some clues to the mechanism underlying these correlations: the overexpression of CD9 rendered cells less susceptible to HIV envelope-mediated syncytia formation [31], the expression rate of CD28 on CD4+ T cells was positively correlated with CD4+ T cell counts [32], while plasma soluble CD27 was inversely correlated to CD4+ T cell counts [33]. A negative correlation between CD52 expression level and CD8+ T cell counts was observed. It has been shown that CD52 expression may be associated with the resting state of T cells [34].

The reliability of the antibody microarray technology was further confirmed by the observation that the CD4+ T cell binding density measured by antibody microarray was significantly correlated with both CD4+ and CD8+ T cell counts measured by flow cytometry, with adjusted p < 0.001 and 0.042, respectively.

Our study is unique in identifying 10 cell surface antigens, whose expression levels distinguished between sustained and transient responder groups, which have implications for the evaluation of HAART efficacy. The mean values for CD11a, CD29, CD38, CD45RO and CD52 binding were significantly higher in the SR group at all time points than those in the TR group, while the mean values for CD56, CD57, CD62E, CD64 and CD33 were significantly lower. Although the biological relevance of the changes observed in these antigens needs further investigation, many of these molecules have already been implicated in HIV infection. CD38 and CD45RO are well documented cell activation markers. CD11a expression on lymphocytes has been shown to be related to clinical stage of disease [35], while CD29 (β-1 integrin chain) is involved in the regulation of an inflammatory effector gene [36]. CD56 is a NK-associated marker and its expression on CD8+ T cells identifies the mature cytolytic effector cells [37,38]. CD57 expression on CD8+, CD4+ T cell and NK cells is a general marker of cell proliferative inability and senescence [39]. CD64 (FcgammaRI) was involved in FcgammaR-mediated phagocytosis, which is impaired by HIV-1 infection in monocyte-derived macrophages [40]. Although the biological roles of CD62E (E-selectin) and CD33 are unknown in the context of HIV infection, the plasma levels of CD62E has been proposed for monitoring disease activity in patients with chronic inflammatory syndromes [41,42] and CD33 expression was significantly increased on alveolar macrophages of HIV+ patients compared with healthy controls [43].

Interestingly, this longitudinal study and our recent cross-sectional study [1] have detected 3 cell surface antigens in common (CD3epsilon, CD9 and CD57). This coincidence may imply that these markers have some crucial roles in HIV disease and HAART. Another notable feature is that both studies have pointed to the importance of cell adhesion molecules in disease progression. Although adhesion molecules have been reported in HIV disease, the biological relevance of most of these molecules is not well understood. Our study provides a strong foundation for understanding biological relevance of most of these molecules through further investigation.

## Conclusion

Our findings not only have implications for the evaluation and future direction of HAART, but also show how in an array-like manner the density of cell surface antigens could be efficiently exploited in studying cell-surface modulation during HAART and HIV-infection. Such investigations would be labor-intensive, time-consuming and expensive if done by flow cytometry. Secondly, the detections of cell surface antigens in our study lay a solid foundation for future functional assessment of these markers. The differential antigens identified in this study should be further investigated by other methods such as flow cytometry for confirmation since DotScan technology does not distinguish between modulation of antigen expression and changes in the proportion of cell population expressing the antigen. A biological analysis of these markers may also help to clarify their role and may lead to the discovery of new biomarkers for HIV prognosis/diagnosis. Further investigation on detailed subset composition of CD4+ and CD8+ T cells should be able to provide more specific information related to immunoreconstitution under therapy since this study cannot differentiate the changes of CD4+ or CD8+ T cell subsets, which may have direct impact on the cell immunophenotype.

## Abbreviations

Abbreviations used in this paper: HAART: Highly active antiretroviral therapy; SR: Sustained responder; TR: Transient responder.

## Competing interests

The authors declare that they have no competing interests.

## Authors' contributions

JQW fully performed the work, analyzed data and wrote the paper. WBD contributed to vital patient samples and immunological interpretation of findings and BW contributed to the writing. LB and JC analyzed data, did statistical evaluation, contributed to the technology and the writing. NKS designed the research project, supervised this work and contributed to the writing. All authors read and approved the final manuscript.
